# Shortcomings in addressing attacks on health care

**DOI:** 10.1186/s13031-018-0182-9

**Published:** 2018-12-11

**Authors:** Dilshad Jaff

**Affiliations:** Research, Innovation and Global Solutions, Gillings School of Global Public Health, University of North Carolina at Chapel Hill, 135 Dauer Drive, Chapel Hill, NC 27599 USA

## Main text

The recent review by Carolyn Briody and colleagues “Review of attacks on health care facilities in six conflicts of the past three decades” is thought provoking and timely [1]. Attacks on healthcare, a strong violation of the Geneva conventions, is appropriately getting more attention and the amount of literature is proliferating steadily. I would like to address two limitations of the article by Briody et al.

First, as the authors note, there is no agreement on the definitions of, for example, “attacks” and “facilities” to ascertain accurate estimates. As a physician in the Iraqi health system and later with the International Committee of the Red Cross (ICRC), I directly experienced frequent attacks on health care facilities and learned of others that were not likely to be included in official reports. The estimation of only 12 incidents between March 2003 to December 2011 cannot possibly reflect the reality and it is misleading to report as such. Further, while entities such as Physicians for Human Rights may include reports in local languages there is no systematic process to gather and analyze these reports, further undermining the credibility of estimates [2].

Second, it is essential to acknowledge that the context in which today’s attacks occur is fundamentally different from in the past. The infrequent examples of such attacks prior to the end of the cold war is likely due to the fact that states were the primary combatants willing to pay at least some attention to the Geneva conventions. The contemporary attacks described by the authors involved state as well as non-state combatants motivated by religion, ethnicity and sect [3]. Given that the world leaders cannot hold state actors accountable, a call for accountability among non-state actors, while desirable, sounds impractical.

## Abbreviation

ICRC: International Committee of the Red Cross
